# P-1461. The Relationship Between Health Insurance Type and Influenza Vaccination Among Pregnant Individuals in the US in 2022 and 2023

**DOI:** 10.1093/ofid/ofaf695.1647

**Published:** 2026-01-11

**Authors:** Jodian A Pinkney, Laura Bogart, Korede Adekanye, Tasmiah Nuzhath, Rocio M Hurtado, Allison Bryant-Mantha, Bisola Ojikutu, Christina Psaros, Ruanne Barnabas

**Affiliations:** Mass General Brigham, Boston, MA; RAND Corporation, Santa Monica, California; Bowen University, Iwo, Osun, Nigeria; University of Alabama, Tuscaloosa, Alabama; Massachusetts General Hospital, Boston, Massachusetts; Massachusetts General Hospital, Boston, Massachusetts; Massachusetts General Hospital, Boston, Massachusetts; Massachusetts General Hospital, Boston, Massachusetts; Massachusetts General Hospital, Boston, Massachusetts

## Abstract

**Background:**

Influenza (flu) infection during pregnancy can lead to adverse maternal and fetal outcomes. Flu vaccination coverage among pregnant individuals remains low, and lack of health insurance may be a modifiable contributing factor to this trend. In 2021, 41.5% of pregnant individuals were covered by Medicaid—a government-sponsored insurance (GSI) that provides healthcare coverage for individuals with low income and specific conditions including pregnancy—while 3.6% were uninsured at the time of delivery.

**Table 1:**
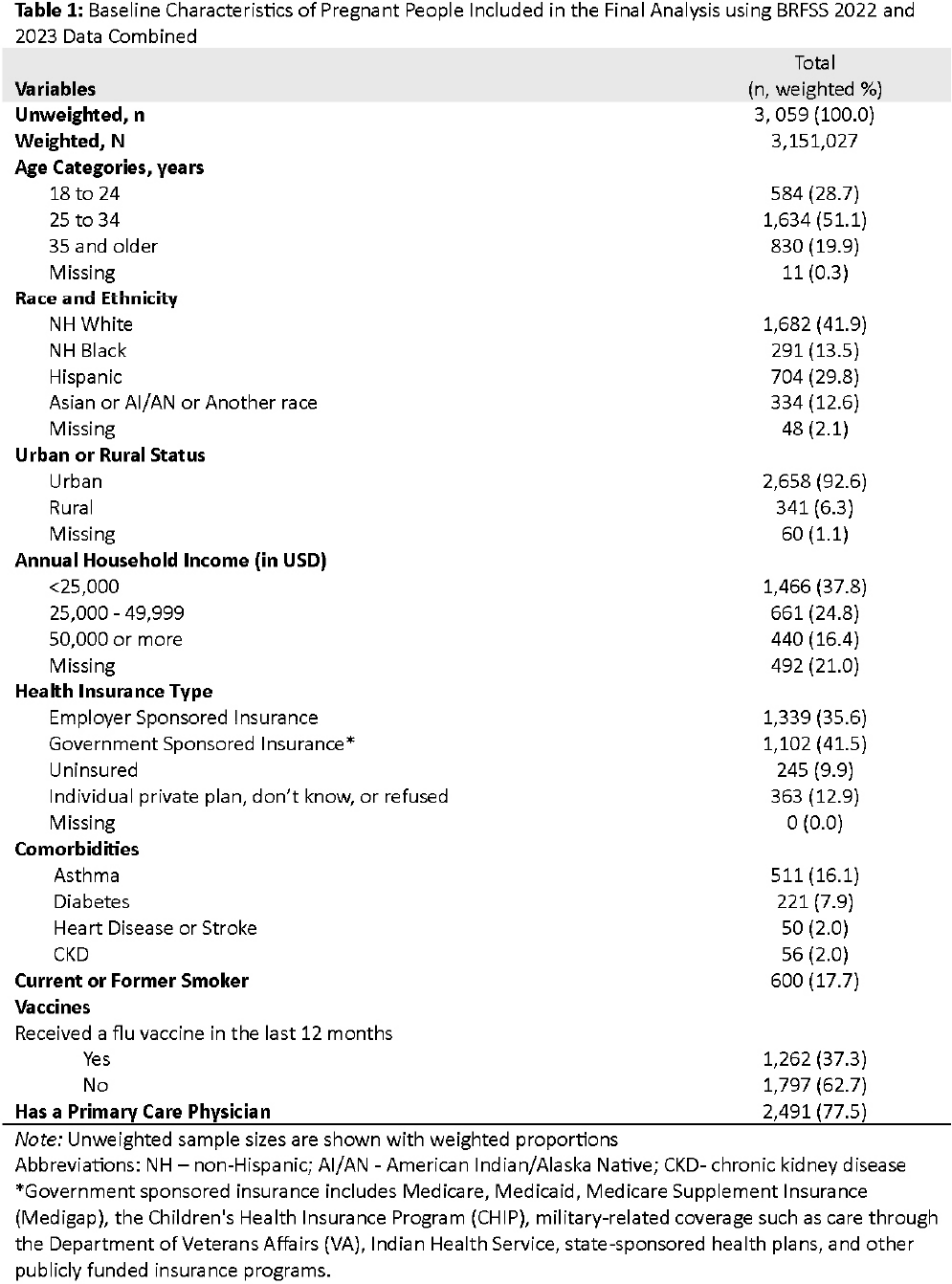
Baseline Characteristics of Pregnant People Included in the Final Analysis using BRFSS 2022 and 2023 Data Combined

**Table 2:**
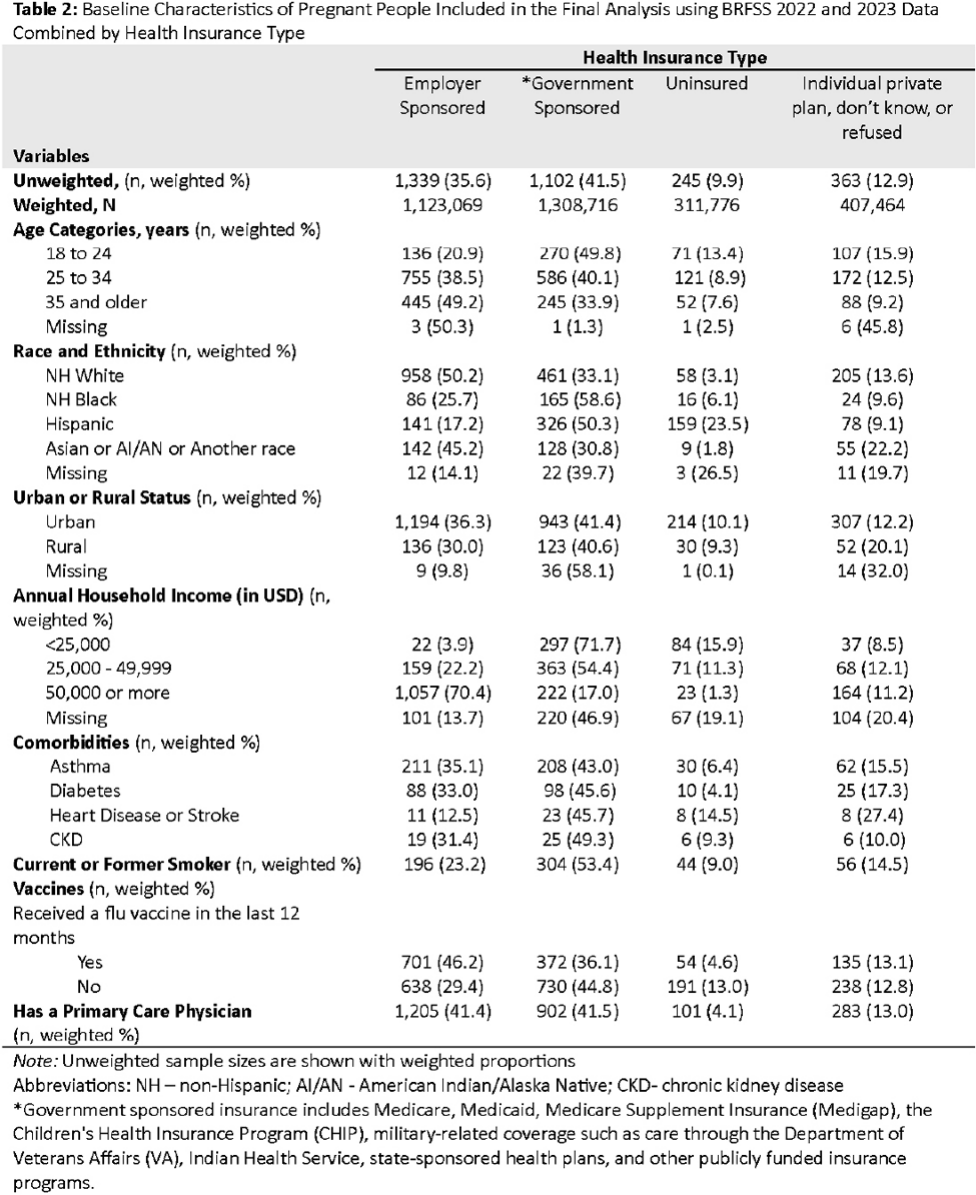
Baseline Characteristics of Pregnant People Included in the Final Analysis using BRFSS 2022 and 2023 Data Combined by Health Insurance Type

**Methods:**

We utilized data from the 2022 and 2023 Behavioral Risk Factor Surveillance System (BRFSS), a national telephone survey in the US, to examine the relationship between health insurance type [employer-sponsored insurance (ESI), GSI, uninsured, or individual private/unknown] and self-reported flu vaccination within the past 12 months among pregnant individuals. Multivariable logistic regression was used to estimate adjusted odds ratios (aORs), controlling for age, income, comorbidities, smoking status, and having a primary care physician (PCP).

**Table 3:**
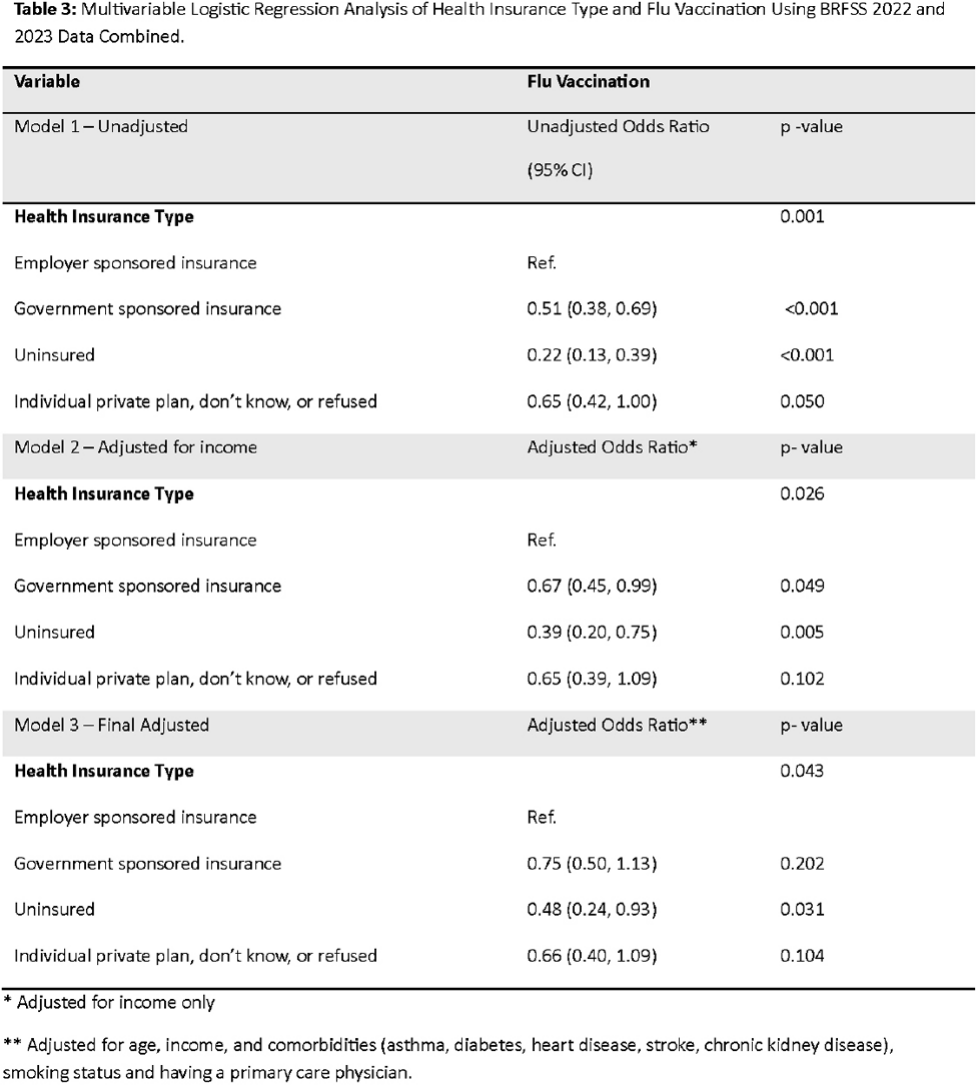
Multivariable Logistic Regression Analysis of Health Insurance Type and Flu Vaccination Using BRFSS 2022 and 2023 Data Combined.

**Results:**

The final analytic dataset included 3,059 pregnant respondents representing a weighted national sample of 3,151,027 pregnant individuals (Table 1). Overall, 37.3% reported receiving the flu vaccine within the last 12 months. Flu vaccination differed by insurance type: 48.4% for ESI, 37.8% for individual private/unknown, 32.5% for GSI, and 17.5% for uninsured individuals. In unadjusted analyses, those with GSI (OR 0.51; 95% CI: 0.38–0.69) and who were uninsured (OR 0.22; 95% CI: 0.13–0.39) had significantly lower odds of vaccination compared to those with ESI (Table 3). After adjusting for covariates, being uninsured remained significantly associated with lower odds of flu vaccination (aOR 0.48; 95% CI: 0.24–0.93).

**Conclusion:**

Pregnant individuals who are uninsured have a lower odds of flu vaccination, likely reflecting decreased access to prenatal care. Preserving access to Medicaid and the Children's Health Insurance Program (CHIP) for eligible pregnant individuals could be an important strategy to improve maternal flu vaccination coverage in the future.

**Disclosures:**

All Authors: No reported disclosures

